# Nutritional Biomarkers and Associated Factors in Community-Dwelling Older Adults: Findings from the SHIELD Study

**DOI:** 10.3390/nu12113329

**Published:** 2020-10-29

**Authors:** Magdalin Cheong, Samuel Teong Huang Chew, Jeffery Oliver, Geraldine Baggs, Yen Ling Low, Choon How How, Ngiap Chuan Tan, Dieu Thi Thu Huynh, Siew Ling Tey

**Affiliations:** 1 Department of Dietetic & Food Services, Changi General Hospital, Singapore 529889, Singapore; magdalin_cheong@cgh.com; 2 Department of Geriatric Medicine, Changi General Hospital, Singapore 529889, Singapore; samuel.chew.t.h@singhealth.com.sg; 3 Yong Loo Lin School of Medicine, National University of Singapore, Singapore 117597, Singapore; 4 Abbott Nutrition Research and Development, Columbus, OH 43219, USA; jeffery.oliver@abbott.com (J.O.); geraldine.baggs@abbott.com (G.B.); 5 Abbott Nutrition Research and Development, Asia-Pacific Center, Singapore 138668, Singapore; yenling.low@abbott.com (Y.L.L.); dieu.huynh@abbott.com (D.T.T.H.); 6 Care and Health Integration, Changi General Hospital, Singapore 529889, Singapore; how.choon.how@singhealth.com.sg; 7 SingHealth-Duke NUS Family Medicine Academic Clinical Program, Duke-NUS Medical School, Singapore 169857, Singapore; tan.ngiap.chuan@singhealth.com.sg; 8 SingHealth Polyclinics, Singapore 150167, Singapore

**Keywords:** nutrition, prevalence, community-dwelling, older adults, elderly, vitamin D, zinc, deficiency, insufficiency

## Abstract

Aging is associated with intrinsic and extrinsic changes which affect the nutrient intake and nutritional status of an older individual. Suboptimal nutritional status is linked with adverse health outcomes. There are limited data in this area for community-dwelling older adults who are not at risk of malnutrition. The objective of this study was to describe the nutritional biomarkers in 400 community-dwelling older adults (aged ≥65 years) with normal nutritional status (Malnutrition Universal Screening Test score of 0) in Singapore and to identify factors associated with these biomarkers. The majority of the participants had normal levels of pre-albumin, albumin, total protein, creatinine, zinc, corrected calcium, vitamin B12, ferritin and hemoglobin. Females had significantly higher levels of corrected calcium and vitamin B12 than males, whereas males had significantly higher levels of pre-albumin, albumin, creatinine, serum ferritin, 25-hydroxyvitamin D (25(OH)D) and hemoglobin than females. About half of the participants (52%) had low level of 25(OH)D (<30 μg/L) and 10% had low zinc level (<724 μg/L). Among those with low level of 25(OH)D, 74% had 25(OH)D insufficiency (20–<30 μg/L) and 26% had 25(OH)D deficiency (<20 μg/L). Younger age, female gender, non-Chinese ethnicity and no intake of vitamin D supplement were associated with lower serum 25(OH)D level, whereas higher body mass index (BMI) was associated with low zinc level. These findings highlight the problem of hidden nutritional insufficiencies can be missed in seemingly normal nourished community-dwelling older adults.

## 1. Introduction

The growth of the aging population worldwide and a longer life expectancy will inevitably have significant healthcare and economic implications. Aging is associated with intrinsic physiological changes, such as poor appetite, absorption capacity and utilization of nutrients, and extrinsic factors, such as eating patterns and socioeconomic factors, which affect nutrient intake and thus the nutritional status of an individual [1,2,3]. Malnutrition in older adults is common, where one in three older adults living independently are reportedly at risk of malnutrition [4]. The prevalence of malnutrition in community-dwelling older adults increases with age [5]. In Asia, studies from China, Taiwan and Japan report that up to 30% of community dwellers are malnourished or at risk of malnutrition, with a higher prevalence in those older than 70 years [6]. In Singapore, the overall prevalence of malnutrition and at risk of malnutrition in community-dwelling older adults (≥55 years) was 2.8% and 27.6%, respectively [7].

Macronutrient and micronutrient inadequacies in community-dwelling older adults are associated with sarcopenia, physical frailty, as well as age-related disorders including cardiovascular disease (CVD) and Alzheimer’s disease [3,8,9,10]. Normal age-related physiological and functional changes also affect nutrient needs; for example, decreased physical activity with advancing age reduces energy requirements, leading to diminished energy and micronutrient intakes [11]. Yet, micronutrient needs in older adults may remain constant or increase [11].

Key micronutrients that have been reported to be inadequate among older adults include calcium, magnesium, potassium, zinc, and vitamins B1, B2, B6, B12, D and E [3,12,13]. Vitamin D inadequacy is common worldwide, especially in older adults [14,15]. Studies in Asia have reported widespread prevalence of vitamin D deficiency and insufficiency in all ages including older adults [16], with deficiency defined as serum vitamin D level <20 μg/L and insufficiency defined as vitamin D level between 20–<30 μg/L [17]. These are important findings because vitamin D deficiency is associated with higher risks of fractures, osteoporosis, and mortality [18,19,20,21]. Older adults are also susceptible to zinc deficiency, as they may reduce consumption of red meat or other zinc-containing food sources, such as shellfish and poultry, to avoid increasing blood cholesterol level, or due to poor dentition and inability to properly masticate food [22,23]. Vitamin B12 deficiency is also reportedly more prevalent in the elderly, possibly due to inadequate intake and poor absorption, and the deficiency generally increases beyond 60 years of age [24,25]. Strict vegetarians, e.g., vegans, who do not consume any animal products are also at risk of vitamin B12 deficiency [25].

Micronutrient deficiencies, as identified from serum levels, have been associated with increasing age, female gender, Malay and Indian ethnicities (compared with Chinese ethnicity), poor eating habits, physical inactivity, smoking, obesity, presence of chronic diseases, intake of medications, no intake of dietary supplements, and low sunlight exposure [26,27,28,29,30,31,32,33,34]. Multiple micronutrient deficiencies may coexist, exacerbating the impact on health outcomes, and concurrent deficiencies in iron, vitamin B12 or folate has been reported in approximately one-third of older adults with anemia [35]. The estimated risk of multiple, concurrent micronutrient deficiencies (2–5 micronutrients) in the US population was 9.4% in adults aged 51–70 years and 12.9% in those ≥71 years [36]. 

Serum biomarkers in general reflect the nutritional status of the individual. Serum proteins, such as pre-albumin and albumin, have been commonly used to determine the nutritional status of clinically stable patients [37,38]. Serum albumin has a long half-life (14–20 days) and is used as a surrogate marker of protein intake [37,38]. Pre-albumin is also used similarly but has a shorter half-life (2–3 days). Hence, it could be a more reliable marker of acute changes of the nutritional state and protein malnutrition [38,39]. One has to be mindful that both markers are negative acute-phase proteins and serum levels are suppressed by inflammation and sepsis. Serum total protein, comprising albumin and globulin, may also provide insights into changes in protein status and has been shown to be a useful biochemical indicator of adult malnutrition [40]. Serum creatinine is a well-known biomarker for skeletal muscle mass as its precursor, creatine phosphate, is stored in the muscle [41]. Serum creatinine is thus a useful biomarker for low muscle mass in individuals with normal renal function [42]. Adequate protein intake and protein distribution are important factors in preventing sarcopenia [43]; sarcopenia may increase the risk of age-related disease and lower the quality of life [44].

Corrected calcium (albumin-adjusted) is used clinically to determine serum ionized calcium levels as serum calcium binds to albumin in the human body, but free ionized calcium is the biologically active form [45]. Serum ferritin level is the most widely used biomarker to determine iron status in clinical and public health settings; it reflects the status of iron stores (iron deficiency inhibits ferritin expression) [46]. Hemoglobin concentration is a reliable indicator of anemia at a population level [47], but is not specific for iron deficiency [48]. Measurement of serum zinc is the most commonly used and accepted biomarker for the determination of zinc status; although hair zinc and urine zinc excretion have been explored, they do not seem to provide greater sensitivity or convenience [48,49]. Zinc has been shown to be essential for maintaining normal immune function; even a mild zinc deficiency may lead to a reduced immune function [50]. Zinc deficiency may also cause dermatitis, diarrhea, depression, impaired taste and decreased appetite [2].

The biologically active form of vitamin D (1,25-hydroxyvitamin D) is not easily measurable. In clinical practice, 25-hydroxyvitamin D [25(OH)D] is a robust biomarker of vitamin D status and has been widely used in population studies of vitamin D inadequacy [51]. A low level of vitamin D in older adults results in reduced mobility and an increased risk for falls and fractures and CVD-associated mortality [52,53]. Vitamin D status also appears to be associated with functional limitations and decline [54] and poor cognitive function [55] in older individuals. Vitamin B12 deficiency may lead to megaloblastic anemia, impaired sensory and peripheral nerve function, bone disease, hearing loss, and macular degeneration in older people, and is a risk factor for cognitive decline and dementia [24,56]. The impact of suboptimal nutritional status on health outcomes, especially in older people, underscores the need for preventive measures in this population.

The nutritional status of older adults is a growing public concern, particularly in light of rapid population aging. Currently, data on the status of nutritional biomarkers in older community-dwellers with normal nutritional status (not at risk of malnutrition) is limited. Availability of such data will help inform public health policy on the need for screening and early detection of nutritional insufficiencies and development of targeted preventive nutrition strategies. Therefore, the objective of this present study was to describe the levels and status of nutritional biomarkers in 400 community-dwelling older adults in Singapore with normal nutritional status (defined as a Malnutrition Universal Screening Test (MUST) score of 0 [57]) and examine factors that influence these nutritional biomarkers.

## 2. Materials and Methods 

### 2.1. Study Design and Participants

Strengthening Health In ELDerly through nutrition (SHIELD) is a cross-sectional study involving community-dwelling older people aged 65 years and above in Singapore. The SHIELD study was conducted in accordance with the ethical principles that have their origins in the Declaration of Helsinki. The study was approved by the SingHealth Centralized Institutional Review Board in Singapore reference number 2017/2273. All enrolled participants provided written informed consent. The study was registered at clinicaltrials.gov as NCT03240952.

Participants were eligible for inclusion in the study based on the following criteria: male or female participant aged ≥65 years; community ambulant with or without aid, screened to have MUST score of 0; community-dweller; had stable chronic disease(s); able to consume food and beverages orally; and able to communicate and follow instructions. People with dementia, type 1 or 2 diabetes, any active infectious disease, severe gastrointestinal disorders, cystic fibrosis, end-stage organ or pre-terminal diseases, acute myocardial infarction within the last 30 days from the screening visit, or active malignancy within the last five years were excluded from the study. MUST was chosen as it is a validated, widely used screening tool for malnutrition in the clinical and community healthcare settings [57,58,59].

### 2.2. Study Procedures

All study participants attended one visit, where socio-demographic information, co-morbidities, malnutrition status, blood samples, anthropometric measurements and body composition were collected. Functional assessment data on physical activity level and activities of daily living were also collected. Socio-demographic data included age, gender, ethnicity, marital status, education, number of prescribed drugs, smoker status and alcohol consumption. The Charlson Comorbidity Index (CCI) was used to determine comorbidity level [60,61]. MUST was used to screen for risk of malnutrition [57]. A nutrition literacy score, consisting of seven questions, was used to assess participant’s nutrition knowledge. Each question had a score of 1 for a correct answer or 0 otherwise. Total score ranged from 0 to 7, with higher score denoting higher nutrition literacy. Data on supplement use were also collected. All supplements used were reviewed, and supplements affecting nutritional biomarkers were identified and reported separately (non-supplement users vs. supplement users). 

Blood samples were collected via venipuncture for the analyses of nutritional biomarkers, such as serum zinc (ICPMS); pre-albumin (COBAS c502); corrected calcium, creatinine, albumin, total protein (COBAS c702); ferritin, 25(OH)D, vitamin B12 (COBAS e801); hemoglobin (Sysmex XN9000). A sub-sample of participants (*n* = 332; 83%) had fasting blood samples for serum zinc analysis. The level of each nutritional biomarker was categorized as low, normal and high. Details are available in Table S1. The level of 25(OH)D was also categorized as deficient <20 μg/L (<20 ng/mL or <50 nmol/L), insufficient 20–<30 μg/L (20–<30 ng/mL or 50–<75 nmol/L) and sufficient ≥30 μg/L (≥30 ng/mL or ≥75 nmol/L) [17].

Standing height was measured without shoes by using a stadiometer (Avamech B1000, Avalanche Mechtronics, Singapore) and body composition such as fat mass, muscle mass and bone mass was measured using bioelectrical impedance analysis (BIA; MC-780, Tanita, Tokyo, Itabashi-Ku). Mid upper arm circumference and calf circumference were measured with an anthropometric tape. Mid upper arm circumference was the mid-point of the anatomical landmarks, the acromion and olecranon. Calf circumference was measured at the largest part of the calf.

Physical activity level was determined using the Physical Activity Scale for the Elderly (PASE) [62,63]. The Modified Barthel Index (MBI) was used to measure functional independence for ten activities of daily living with dependency level score ranging from 0–20 as total dependent, 21–59 as dependent, 60–79 as moderate dependent, 80–99 as slight dependent, and 100 as independent [64].

### 2.3. Data Analysis

Baseline characteristics of the study participants were reported as means and standard error for continuous variables, and numbers and percentages for categorical variables. For continuous variables, normality of the data was assessed using the Shapiro–Wilk test (*p* < 0.001) and graphical methods.

The levels of nutritional biomarkers were summarized by gender and ethnicity, as the reference ranges or cut-offs for some of the biomarkers are impacted by gender or ethnicity. Use of supplements are known to significantly impact certain nutritional biomarkers, and as such a comparison between non-supplement users and supplement users was performed. 

Multiple linear regression models were used to examine the associations between nutritional biomarkers and all identified variables based on the literature in this area and clinical relevance. These include known factors affecting specific nutritional biomarkers, surrogate measures of nutritional status, socio-economic status, and supplement usage. In this study, 106 participants did not have detectable C reactive protein due to their normal nutritional status and 98% of the total cohort had a Charlson comorbidity score of 0. Only participants with measurable C reactive protein values were included in the multiple linear regression models (*n* = 294). The residuals from the linear models of each biomarker were checked for normality using a Shapiro–Wilk *p* value. Multicollinearity between the predictors was tested using variance inflation factor and tolerance. 

All statistical analyses were performed using SAS version 9.4 (SAS Institute, Cary, NC, USA). *p* < 0.05 was considered statistically significant.

## 3. Results

### 3.1. Baseline Clinical Characteristics and Nutritional Biomarker Levels of Study Participants

As shown in Table 1, a total of 400 individuals participated in the study, including 183 (45.75%) men and 217 (54.25%) women. The mean age of study participants was 71.21 ± 0.26 years, with a mean body mass index (BMI) of 24.53 ± 0.15 kg/m^2^. Of the study participants, 98% had a Charlson Comorbidity score of 0 and 93% were physically independent (Modified Barthel Index score of 100). Gender differences were observed for height, body weight, calf circumference, fat mass, body fat percentage, muscle mass, ethnicity, nutrition literacy score, education level, smoking status and alcohol consumption (Table 1; all *p* ≤ 0.0036). 

The levels of corrected calcium and vitamin B12 were significantly higher in women than in men, whereas the levels of pre-albumin, albumin, creatinine, serum ferritin, 25(OH)D and hemoglobin were significantly higher in men than in women (Table 1; all *p* ≤ 0.0232). No gender differences were observed in the levels of zinc and total protein.

Over 90% of participants had normal levels of pre-albumin, albumin, total protein, creatinine, corrected calcium and vitamin B12 (Table 2). Almost three quarters (73%) of participants had a normal ferritin level and 27% had a high ferritin level. Similarly, 81% had a normal hemoglobin level and 16% had a high hemoglobin level. Overall, 89% of participants had a normal zinc level but 10% had a low zinc level. More than half of total study participants (*n* = 208; 52%) had a low level of 25(OH)D; more women had a low level of 25(OH)D (Table 2). Out of these 208 participants, 54 (26%) of them had 25(OH)D deficiency and 154 (74%) had 25(OH)D insufficiency (Table S2). 

There was a statistically significant association between gender and the levels of creatinine, corrected calcium, serum ferritin and hemoglobin (Table 2; all *p* ≤ 0.0459). Compared to women, there were a higher number of men with a high level of creatinine (7% versus 3%), serum ferritin (41% versus 14%) and hemoglobin (29% versus 4%). On the other hand, more men had a low corrected calcium level (7% versus 2%) than women. Compared with other ethnicities, a higher percentage of the Chinese population were within the normal range for serum total protein, 25(OH)D, zinc, and hemoglobin (all *p* ≤ 0.0386). 

### 3.2. Associations between Nutritional Biomarkers and Various Factors (Pearson Correlation and Analysis of Variance (ANOVA))

As shown in Table 3, age was positively correlated to 25(OH)D and creatinine levels were negatively correlated to pre-albumin and hemoglobin levels (all *p* ≤ 0.0066). BMI was negatively associated with albumin and zinc levels whereas it was positively associated with corrected calcium (all *p* ≤ 0.0167). Calf circumference was positively correlated with creatinine and hemoglobin levels (all *p* ≤ 0.0305). C-reactive protein was negatively associated with pre-albumin and albumin levels while positively associated with creatinine (all *p* ≤ 0.0311).

Of the 400 study participants, 88 took a calcium supplement, 77 took a vitamin D supplement, 57 took a vitamin B12 supplement, and 11 took an iron supplement that might affect both ferritin and hemoglobin levels. Vitamin D and vitamin B12 supplements users had higher levels of 25(OH)D and vitamin B12 than non-supplement users (Table 4; both *p* ≤ 0.0002). On the other hand, iron supplement users had a lower hemoglobin level compared with non-iron supplement users (Table 4; *p* < 0.0001).

When comparing the results of non-supplement taking men and women, men had lower corrected calcium and vitamin B12 than women (Table 4; both *p* ≤ 0.0192) whereas men had higher 25(OH)D, ferritin and hemoglobin than women (all *p* ≤ 0.0020). There were no significant differences in any of the nutritional biomarkers between supplemented men and women (Table 4; all *p* ≥ 0.0532), which may be due to small sample sizes. 

As shown in Table S2, the prevalence of vitamin D insufficiency (20–<30 μg/L) was 38.5%, and 13.5% of participants had vitamin D deficiency (<20 μg/L). Significant associations were observed between the nutritional status (deficient, insufficient, sufficient) of vitamin D and age, calf circumference, ethnicity, drinking habit, supplement use, eating less due to poor vision, and ability to shop, cook and feed oneself (Table S2; all *p* ≤ 0.0491). Participants with a sufficient level of vitamin D were older than those with vitamin D deficiency and insufficiency. On the other hand, participants with vitamin D deficiency had lower calf circumference than those with vitamin D insufficiency and sufficiency. About 10% of participants had zinc deficiency (<724 μg/L) based on fasting level. The nutritional status of zinc was significantly associated with BMI, ethnicity, body weight, calf circumference, Modified Barthel Index score, total Charlson Comorbidity score, smoking, hospital admission in the last 6 months, eating less due to reduced taste sensation, eating less due to poor vision, and ability to shop, cook and feed oneself (Table S2; both *p* ≤ 0.0481). Participants with a low zinc level had higher BMI than those with normal zinc level. 

### 3.3. Factors Associated with Nutritional Biomarker Levels in Study Participants (Multivariable Models)

Women were significantly associated with higher level of corrected calcium compared with men, whereas men were associated with higher levels of 25(OH)D, serum ferritin, hemoglobin, pre-albumin and creatinine (Table 5; all *p* ≤ 0.0179). Age was positively associated with 25(OH)D and creatinine levels while being negatively associated with hemoglobin and pre-albumin levels (all *p* ≤ 0.0212). Chinese ethnicity was associated with higher levels of 25(OH)D, serum ferritin and hemoglobin compared with the non-Chinese ethnicity group (all *p* ≤ 0.0274). BMI was positively associated with hemoglobin level whereas it was negatively associated with the levels of albumin and zinc (all *p* ≤ 0.0343). The use of vitamin D supplement and vitamin B12 supplement was also associated with higher levels of 25(OH)D and vitamin B12, respectively, compared with no supplements use (both *p* ≤ 0.0036). The use of iron supplement was associated with lower hemoglobin level than no supplement use (*p* < 0.0001).

## 4. Discussion

To the best of our knowledge, this is the first cross-sectional study that examines the levels of various nutritional biomarkers of community-dwelling older adults with normal nutritional status in Singapore, providing us with a unique set of data in this cohort. Our study found that a majority of the community-dwelling older people had normal levels of vitamin B12, pre-albumin, albumin, total protein, creatinine, zinc, corrected calcium, serum ferritin and hemoglobin. However, 52% had low serum 25(OH)D (<30 μg/L), with 13.5% and 38.5% demonstrating vitamin D deficiency and vitamin D insufficiency, respectively. Furthermore, 10% of the community-dwelling older people had a low zinc level (<724 μg/L). Multivariable regression analyses subsequently identified factors that were independently associated with the levels of nutritional biomarkers. Younger age, female gender, non-Chinese ethnicity and no intake of vitamin D supplement were associated with lower serum 25(OH)D level, whereas higher BMI was associated with low zinc level.

### 4.1. Nutritional Biomarkers and Associated Factors in Community-Dwelling Older Adults

#### 4.1.1. Vitamin D

In line with previous studies [26,27,29,65,66,67,68], our results also showed a high prevalence of suboptimal vitamin D, despite a normal nutritional status as screened by MUST, in community-dwelling older adults in Singapore. As 30.4% of older adults are at risk of malnutrition or being malnourished in Singapore [7], the prevalence of vitamin D deficiency and insufficiency is likely to be higher in the general population of older adults. Furthermore, our data are consistent with other Asian studies, where a high prevalence of vitamin D deficiency <20 μg/L (<20 ng/mL or <50 nmol/L) and insufficiency 20–<30 μg/L (20–<30 ng/mL or 50–<75 nmol/L) has been reported in Asian elderly aged ≥ 60 years; 34.1–74.7% Chinese men and 44.0–78.5% Chinese women had serum 25(OH)D <20 ng/mL [26,66,68], and 30.6% Taiwanese men and 57.7% Taiwanese women aged ≥65 years had serum 25(OH)D <30 ng/mL [69]. 

In our study cohort, age was positively associated with 25(OH)D. This is in line with the association reported in another study in a Singapore population [31]. This positive association may be attributed to the significantly higher rates of vitamin D supplementation in older adults in the present study, where 16.7% of the participants aged 65–74 years took vitamin D supplements, compared to 23.7% among participants aged ≥75 years (*p* = 0.0152; data not shown). The other contributory factor could be the additional time spent in outdoor activities with increased sun exposure post-retirement [16]. A Dutch study of older adults aged 60–87 years reported an inverse relationship between age and 25(OH)D level [34]; however, other studies did not find any significant associations between age and the level of 25(OH)D in their study cohorts [26,69,70]. The possible explanations offered in the literature, which also apply to our study cohort include the use of supplements in the older population and the increase in sun exposure as a result of increased leisure time from retirement. The latter may be more significant in the Singapore population at the equatorial latitude as compared to population in higher or lower latitude regions.

An inverse relationship between female gender and the level of 25(OH)D has been reported in several studies [26,31,34,69,71,72,73]. In line with previous studies, the present study shows that female gender was associated with lower level of 25(OH)D. Women generally have a higher body fat percentage compared with men [26,31,34]; as vitamin D can be stored in fat, the serum 25(OH) level in women may be lower. Cultural or religious values may also play an important role; for example, Asian women tend to favor lighter skin color and may, therefore, avoid sun exposure or use sunscreen more frequently than men [31,69,74]. Older Taiwanese women with low and moderate sun exposure were found to be at a greater risk of low vitamin D status compared with those with high sun exposure [69]. Traditional religious attire worn by some women may only expose the face and hands, thus limiting sun exposure [31,75]. 

Ethnic differences have also been shown to be a determining factor for vitamin D level [31,67,76]. Consistent with previous studies reporting this observation, we found that other ethnic groups were associated with lower level of 25(OH)D compared with individuals of Chinese ethnicity. Absorption of sunlight is inversely correlated with skin pigmentation [77]. Furthermore, vitamin D synthesis was also inhibited in individuals with darker pigmentation and this is compounded by traditional attire preventing exposure to sunlight. As such, this may explain the higher risk of vitamin D insufficiency and deficiency in other ethnic groups.

The vitamin D level in older adults was significantly influenced by intake of vitamin D supplements [26,27,34,65,70]. Similarly, we found that intake of vitamin D supplements was associated with higher serum 25(OH)D level. Regular supplementation with vitamin D has been shown to be effective in improving serum 25(OH)D level in adults above 19 years of age [78]. Hence, once vitamin D deficiency or insufficiency is identified, it can be effectively treated with oral supplementation.

#### 4.1.2. Zinc

In our study, a low level of fasting serum zinc (<724 μg/L) was found in 10% of the study participants, with no difference between genders. The reported level of zinc deficiency from other available studies varied, with notable differences in cutoff values and methods of measurement.

The prevalence of serum zinc deficiency observed in the present study was in line with a previous study in older individuals at low risk of malnutrition (MUST score = 0) in Norway, which reported a prevalence of serum zinc deficiency of 12% in men and 6.7% in women. In the same study, the prevalence was significantly higher in individuals at medium or high risk of malnutrition (MUST score = 1 or 2; 31.7% in men and 12.3% in women) [79]. Hence, the true prevalence of zinc deficiency is also likely to be higher in the general population aged 65 and above in Singapore, as this would include those at risk of malnutrition.

Previous literature suggests that increasing age is associated with an increasing prevalence of zinc deficiency. In the Norwegian study described above, prevalence of zinc deficiency increased with advancing age, from 8.9% in the age range of 64–71 years to 16.2% in the age range of 79–87 years [79]. An older study conducted in five European countries found that 31% of people over 60 years of age had zinc deficiency (≤719 μg/L) [80]. A study in a Japanese population found that the prevalence of zinc deficiency, based on scalp hair zinc concentration, increased with aging; from 11.6% (60–69 years) to 15.1% (70–79 years) in men, and from 8.5% (60–69 years) to 15.4% (70–79 years) in women [81]. In the present study, participants with zinc deficiency were older versus those with normal zinc level, although this comparison did not reach statistical significance. This could be due to the smaller sample size and narrower age range in this study compared with the previous studies.

In our study, we found that low zinc level was associated with higher BMI, which was consistent with other studies. Serum zinc level has been reported to be lower in overweight or obese individuals than in those with normal BMI [82,83,84,85]. Reduction of body weight and BMI resulted in an increase of serum zinc level [86]. The underlying mechanisms for serum zinc level and obesity are not well understood but several explanations have been proposed. The presence of chronic inflammation and stress in obesity induces the expression of metallothionein and zinc transporters, which promote the absorption of zinc by adipocytes, thereby reducing serum or plasma concentration [82]. Zinc deficiency can also result in the release of reactive oxygen species in tissues leading to oxidative stress, which in turn results in obesity-related complications [82]. In the presence of low dietary zinc intake, obese individuals are also less capable of responding to oxidative stress and inflammation, which then worsens the obesity status [87]. Furthermore, low serum zinc level is associated with an elevated level of circulating leptin which causes leptin resistance in obesity due to impairments in the leptin-signaling pathway [82]. Leptin also promotes the expression of inflammatory cytokines, resulting in persistent inflammation and the development of obesity-related inflammatory states in obese individuals [82].

#### 4.1.3. Hemoglobin and Ferritin 

A majority of the community-dwelling older adults in the present study had normal levels of hemoglobin and ferritin. US national data showed that 11.0% of men and 10.2% of women ≥65 years of age had low level of hemoglobin (anemia; World Health Organization (WHO) cut-off: men <130 g/L and women <120 g/L) [35]. A systematic review also reported that the prevalence of anemia (WHO cut-off) in community-dwelling older adults (≥65 years) was 12% [88]. Among Chinese rural residents surveyed in China National Nutrition and Health Survey, the prevalence of anemia in adults ≥60 years of age was 12.6% (WHO cut-off, with values adjusted to account for long-term altitude exposure) [89]. A recent study in Malaysian elderly (≥60 years) reported a much higher prevalence of anemia (35.3%; WHO cut-off) in this population, with the highest prevalence found in participants of Indian ethnicity (45.5%) [90]. The authors suggested that this could be partly due to the high prevalence of diabetes mellitus and chronic kidney disease in the total cohort, with vegetarian diet playing a part in the Indian population. 

In our study, only 4% of participants had a low level of hemoglobin based on the local hospital laboratory cut-off of <11.5 g/dL for both men and women. Upon further analysis using WHO cut-off, we found a prevalence of 11% for low hemoglobin in the total cohort, 13% for men and 9% for women (*p* = 0.2617) (data not shown). This is in line with previous community studies in US and China. In addition, our study found a significantly higher prevalence of low hemoglobin among non-Chinese (19%) compared to Chinese (9%) (*p* = 0.0309) (data not shown), in keeping with the Malaysian study. These results derive from a cohort with normal nutritional status, very low comorbidities and high function suggesting a higher prevalence in the general population, hence warranting more attention with regards to screening and management of anemia in the community setting in Singapore, particularly in the non-Chinese group.

In our study, male gender was associated with high levels of hemoglobin (>15 g/dL) and serum ferritin (>294 μg/L). Evidence suggests that smoking can influence hemoglobin level. Elevated serum ferritin was observed in former or current smokers in the Korean adult population (≥40 years) [91]. A retrospective Danish study including individuals aged 30–60 years found that smokers had significantly higher mean of hemoglobin compared to non-smokers regardless of gender [92]. In our study, more men than women were past smokers (26.2% versus 4.1%) or daily/occasional smokers (4.9% versus 0.5%). This may potentially explain the higher proportion of men with high levels of hemoglobin and ferritin (29% and 41%, respectively) versus women (4% and 14%, respectively) in our study. The majority of women in our study had normal levels of hemoglobin and ferritin (91% and 85%, respectively); however, it is noted that 5% of women had low hemoglobin level (<11.5 g/dL). Heavy menstrual bleeding has been reported to affect up to ~15–30% of pre-menopausal women, which is a common cause of iron deficiency anemia [93,94]. However, ~45–80% of women experiencing heavy menstrual bleeding did not seek medical attention [93,94], and may thus risk sustaining undiagnosed anemia into older age [95].

#### 4.1.4. Other Biomarkers (Calcium, Vitamin B12, Pre-Albumin, Albumin) 

Most community-dwelling older people in our study had normal levels of calcium, vitamin B12, pre-albumin, and albumin; low levels of these biomarkers were seen in ≤5% of our study population. This is because all study participants had normal nutritional status and majority of the study cohort (98%) had a Charlson Comorbidity score of 0.

In our study, male gender was associated with lower level of corrected calcium. In a study of a Norwegian population, serum calcium in men exhibited a declining trend with increasing age (from 2.41 mmol/L in their 20s to 2.34 mmol/L in their 80s); in women, serum calcium was stable before menopause (2.35 mmol/L) and subsequently plateaued at 2.39 mmol/L [96]. Although there was a gender difference in the corrected calcium level (2.21 mmol/L in men vs. 2.25 mmol/L in women), both values lie within the normal range (2.10–2.60 mmol/L) and, therefore, this should not have an impact on normal physiological function. 

The prevalence of vitamin B12 deficiency based on US national data (<148 pmol/L) was approximately 6% in adults ≥70 years of age [97]; several UK population surveys reported a prevalence of vitamin B12 deficiency (≤150 pmol/L) of around 5% in adults aged 65–74 years and at least 10% in adults ≥75 years of age [97]. In the present study, 2% had low level of vitamin B12 (<132 pmol/L). However, the true prevalence of vitamin B12 deficiency among the older adults in Singapore is likely to be higher, as our study cohort excluded individuals with type 2 diabetes, and there is a known association between the use of metformin and vitamin B12 deficiency in people with diabetes [98,99]. 

In a small sample of community-dwelling older adults in Japan, 11.1% of the study population had low levels of pre-albumin (<21.0 mg/dL) and 6.3% had low levels of albumin (<35.0 mg/dL) [100]. A larger study of community-dwelling Japanese older adults (*n* = 99) reported that prevalence of low albumin level (≤35.0 mg/dL) was about 5% [101]. In our study, 5% had low pre-albumin level (<20 mg/dL) and none of the participants had low albumin level (<37 mg/dL). The lower prevalence observed in this study could be due to the good nutritional status of the participants [79,80,81].

### 4.2. Public Health Measures

One of the challenges in developing effective public health policies for healthy older adults is the lack of clinically relevant data. The present study was designed to address this need and give a robust set of data to guide policy development and also for future comparisons with other studies in similar cohorts. 

Our study found a high prevalence of suboptimal vitamin D and presence of zinc deficiency among community-dwelling older adults with normal nutritional status in Singapore. Given that our study cohort of older adults had normal nutritional status, the actual prevalence of vitamin D inadequacy and low zinc level among older adults is likely to be higher than that observed in our study. These findings are important because inadequate vitamin D may result in reduced mobility, increased risk for falls, and functional and cognitive decline in older adults [52,53,54,55], while zinc deficiency may lead to a reduced immune function, delayed wound healing, and affect taste perception and contribute to appetite loss and anorexia [50,102,103,104].This is a public health concern in light of the rapid population aging in Singapore [105] and in spite of ongoing efforts to promote optimal vitamin D levels [106,107]. The high prevalence of suboptimal vitamin D status among older adults with normal nutritional status residing in a sunny tropical region in this study highlights the need to increase awareness among clinicians on this condition. 

Population screening programs were found to be cost-effective, particularly in those aged over 70 years and those at high-risk of fractures [108,109]. In older people who are at greater risk of having suboptimal vitamin D status, it is important to increase sun exposure and promote vitamin D rich foods and supplementation as a means to improve vitamin D status [26,66,69,78]. Furthermore, promoting the use of supplements may be an easy, inexpensive and effective strategy to prevent micronutrient deficiency [65].

Our study identified factors associated with suboptimal levels of vitamin D in community-dwelling older adults, which could help inform those who would benefit most from screening. These significant results could be used to inform government or healthcare institutions in implementing targeted public health policies to address nutritional insufficiencies or deficiencies. The potential benefits are reduction of diet-associated diseases, improving clinical outcomes and reducing healthcare resource utilization.

### 4.3. Study Limitations

This study is not without limitations. Causal relationships between suboptimal nutritional biomarkers and factors associated with it could not be established due to the cross-sectional nature of the study. Future prospective studies are, therefore, needed to confirm the association identified in this study. In addition, dietary intake data were not collected, and future research could examine their relationship with nutritional biomarkers in this cohort. Nevertheless, this study is unique as it is one of the first studies to profile the status of nutritional biomarkers and investigate the associated factors in community-dwelling older adults with normal nutritional status. This is important as this group of people are often perceived to be healthy and are not targeted for screening of nutritional deficiencies. Further research is warranted to identify the underlying mechanisms and quantify the impact of lifestyle and dietary interventions on nutrient deficiencies in older adults, as a follow up of the findings from this study. 

## 5. Conclusions

In conclusion, our findings are clinically important and relevant in light of the increasing aging populations worldwide. Our study found a high prevalence of suboptimal vitamin D and presence of zinc deficiency among community-dwelling older adults with normal nutritional status. These findings deserve urgent attention and highlight the need for early detection of nutritional insufficiencies in older people and could be used as a guide in the development of targeted preventive public health nutrition strategies.

## Figures and Tables

**Table 1:** Baseline characteristics of study participants.

Baseline Characteristics	Total (*n* = 400)	Men (*n* = 183)	Women (*n* = 217)	*p* Value ^a^
Age (years)	71.21 (0.26)	71.26 (0.38)	71.18 (0.37)	0.8774
Height (cm)	158.68 (0.43)	165.57 (0.45)	152.87 (0.36)	<0.0001
Body weight (kg)	61.86 (0.48)	66.75 (0.68)	57.74 (0.54)	<0.0001
BMI (kg/m^2^)	24.53 (0.15)	24.31 (0.21)	24.71 (0.22)	0.1966
BMI categories, *n* (%)				
Normal (18.5–24.9 kg/m^2^)	245 (61.25)	121 (66.12)	124 (57.14)	0.0514
Overweight (25–29.99 kg/m^2^)	134 (33.50)	57 (31.15)	77 (35.48)	
Obese (≥30 kg/m^2^)	21 (5.25)	5 (2.73)	16 (7.37)	
Mid upper arm circumference (cm)	27.73 (0.16)	27.99 (0.22)	27.51 (0.24)	0.1474
Calf circumference (cm)	35.24 (0.16)	36.02 (0.23)	34.59 (0.21)	<0.0001
Fat mass (kg)	17.87 (0.32)	14.79 (0.39)	20.43 (0.42)	<0.0001
Fat (%)	28.50 (0.45)	21.60 (0.41)	34.25 (0.47)	<0.0001
Muscle mass (kg)	41.57 (0.41)	49.14 (0.42)	35.25(0.22)	<0.0001
Ethnicity, *n* (%)				
Chinese	332 (83.0)	141 (77.0)	191 (88.0)	0.0036
Non-Chinese	68 (17.0)	42 (23.0)	26 (12.0)	
Nutrition literacy score, *n* (%)				<0.0001
2 or 3	5 (1.3)	2 (1.1)	3 (1.4)	
4 or 5	180 (45.0)	104 (56.8)	76 (35.0)	
6 or 7	215 (53.8)	77 (42.1)	138 (63.6)	
Highest level of education, *n* (%)				
No formal education or primary level	62 (15.5)	18 (9.8)	44 (20.3)	0.0001
Secondary O/N level or equivalent	186 (46.5)	75 (41.0)	111 (51.2)	
A level or equivalent	100 (25.0)	60 (32.8)	40 (18.4)	
University and above	52 (13.0)	30 (16.4)	22 (10.1)	
Housing type, *n* (%)				0.1304
HDB 1–3 room flats	74 (18.5)	39 (21.3)	35 (16.1)	
HDB 4–5 room flats	186 (46.5)	89 (48.6)	97 (44.7)	
Private properties + others	140 (35.0)	55 (30.1)	85 (39.2)	
Smoking status, *n* (%)				
Non-smoker	333 (83.25)	126 (68.9)	207 (95.4)	<0.0001
Past smoker	57 (14.25)	48 (26.2)	9 (4.1)	
Daily/occasional smoker	10 (2.5)	9 (4.9)	1 (0.5)	
Alcohol consumption ^b^, *n* (%)				
No alcohol	287 (71.75)	119 (65.0)	168 (77.4)	0.0034
<Once a month	69 (17.25)	34 (18.6)	35 (16.1)	
≥Once a month	44 (11.00)	30 (16.4)	14 (6.5)	
Physical Activity Scale for the Elderly score	119.45 (3.17)	122.86 (5.14)	116.58 (3.93)	0.3240
Modified Barthel Index score	99.54 (0.12)	99.43 (0.21)	99.64 (0.13)	0.3831
Modified Barthel Index, *n* (%)				
Moderate dependence (60–79)	7 (1.8)	4 (2.2)	3 (1.4)	0.6737
Slight dependence (80–99)	21 (5.3)	11 (6.0)	10 (4.6)	
Independent (100)	372 (93.0)	168 (91.8)	204 (94.0)	
Total Charlson Comorbidity Score	0.03 (0.01)	0.04 (0.02)	0.02 (0.01)	0.3623
Charlson Comorbidity Score, *n* (%)				
0	392 (98.0)	178 (97.3)	214 (98.6)	0.3757
1	6 (1.5)	4 (2.2)	2 (0.9)	
2	1 (0.3)	0 (0.0)	1 (0.5)	
3	1 (0.3)	1 (0.5)	0	
Pre-albumin (mg/dL)	26.91 (0.26)	28.34 (0.37)	25.70 (0.35)	<0.0001
Albumin (mg/dL)	45.9 (0.1)	46.2 (0.2)	45.6 (0.2)	0.0232
Total protein (g/L)	71.5 (0.2)	71.4 (0.3)	71.6 (0.3)	0.4908
Creatinine (μmol/L)	79.6 (1.3)	96.8 (2.1)	65.0 (0.8)	<0.0001
25(OH)D (μg/L)	30.42 (0.51)	31.87 (0.79)	29.19 (0.65)	0.0084
Vitamin B12 (pmol/L) ^c^	423.4 (9.7)	395.2 (12.3)	447.1 (14.3)	0.0072
Zinc (μg/L) ^d^	862.4 (7.0)	865.5 (12.1)	859.9 (8.2)	0.6912
Corrected calcium (mmol/L)	2.232 (0.004)	2.212 (0.006)	2.249 (0.006)	<0.0001
Serum ferritin (μg/L)	249.20 (7.95)	283.49 (13.12)	220.28 (9.19)	<0.0001
Hemoglobin (g/dL)	13.73 (0.07)	14.34 (0.1)	13.22 (0.07)	<0.0001

BMI, body mass index. O/N level, Ordinary/Normal level. A level, Advanced level. HDB, housing development board. 25(OH)D, 25-hydroxyvitamin D. All values are mean (SE), unless otherwise specified. ^a^ Analysis of variance; *p* value significance <0.05. ^b^ Consumption in the last 12 months. ^c^ Total participants (*n*) = 396 (men: 181; women: 215). ^d^ Total participants (*n*) = 332 (men: 149; women: 183).

**Table 2:** Nutritional biomarkers status by gender and ethnicity.

Nutritional Biomarkers	Status	Total (*n* = 400)	Men (*n* = 183)	Women (*n* = 217)	*p* Value ^a^	Chinese (*n* = 332)	Non-Chinese (*n* = 68)	*p* Value ^a^
Pre-albumin (mg/dL)	Low (<20)	19 (5)	4 (2)	15 (7)	0.0515	14 (4)	5 (7)	0.3483
	Normal (20–40)	373 (93)	174 (95)	199 (92)		312 (94)	61 (90)	
	High (>40)	8 (2)	5 (3)	3 (1)		6 (2)	2 (3)	
Albumin (mg/dL)	Low (<37)	0	0	0	1.0000	0	0	0.0762
	Normal (37–51)	397 (99)	182 (99)	215 (99)		331 (>99)	66 (97)	
	High (>51)	3 (1)	1 (1)	2 (1)		1 (<1)	2 (3)	
Total protein (g/L)	Low (<62)	2 (1)	2 (1)	0	0.2087	0	2 (3)	0.0285
	Normal (62–82)	398 (100)	181 (99)	217 (100)		332 (100)	66 (97)	
	High (>82)	0	0	0		0	0	
Creatinine (μmol/L)	Low (F: <50; M: <65)	13 (3)	3 (2)	10 (5)	0.0353	13 (4)	0	0.2583
	Normal (F: 50–90; M: 65–125)	368 (92)	167 (91)	201 (93)		303 (91)	65 (96)	
	High (F: >90; M: >125)	19 (5)	13 (7)	6 (3)		16 (5)	3 (4)	
25(OH)-D (μg/L)	Low (<30)	208 (52)	88 (48)	120 (55)	0.1605	162 (49)	46 (68)	0.0051
	Normal (30–100)	192 (48)	95 (52)	97 (45)		170 (51)	22 (32)	
	High (>100)	0	0	0		0	0	
Vitamin B12 (pmol/L) ^b^	Low (<132)	7 (2)	2 (1)	5 (2)	0.2720	6 (2)	1 (2)	1.0000
	Normal (132-835)	372 (94)	174 (96)	198 (92)		310 (94)	62 (94)	
	High (>835)	17 (4)	5 (3)	12 (6)		14 (4)	3 (5)	
Zinc (μg/L) ^c^	Low (<724)	34(10)	16 (11)	18 (10)	0.7338	23 (8)	11 (20)	0.0386
	Normal (724–1244)	295 (89)	131 (88)	164 (90)		250 (91)	45 (80)	
	High (>1244)	3 (1)	2 (1)	1 (1)		3 (1)	0	
Corrected calcium (mmol/L)	Low (<2.10)	17 (4)	12 (7)	5 (2)	0.0459	14 (4)	3 (4)	1.0000
	Normal (2.10-2.60)	383 (96)	171 (93)	212 (98)		318 (96)	65 (96)	
	High (>2.60)	0	0	0		0	0	
Serum ferritin (μg/L)	Low (F: <18.2; M: <32)	2 (1)	1(1)	1 (<1)	<0.0001	2 (1)	0	0.2484
	Normal (F: 18.2–339; M: 32–294)	292 (73)	107 (58)	185 (85)		237 (71)	55 (81)	
	High (F: >339; M: >294)	106 (27)	75 (41)	31 (14)		93 (28)	13 (19)	
Hemoglobin (g/dL)	Low (<11.5)	16 (4)	5 (3)	11 (5)	<0.0001	9 (3)	7 (10)	0.0066
	Normal (11.5–15.0)	322 (81)	125 (68)	197 (91)		275 (83)	47 (69)	
	High (>15.0)	62 (16)	53 (29)	9 (4)		48 (14)	14 (21)	

25(OH)D, 25-hydroxyvitamin D. F, female. M, male. Values presented are number of participants (%). ^a^ Fisher’s exact test; *p* value significance <0.05. ^b^ Total participants (*n*) = 396 (men: 181; women: 215). ^c^ Total participants (*n*) = 332 (men: 149; women: 183).

**Table 3:** Correlations of various factors with nutritional biomarkers *.

Nutritional Biomarkers	Age	BMI	Calf Circumference	C-Reactive Protein
	(year)	(kg/m^2^)	(cm)	(mg/L)
Pre-albumin				
Correlation	−0.136	−0.017	−0.017	−0.219
*p* value	0.0066	0.7363	0.7325	0.0001
Albumin				
Correlation	−0.062	−0.162	−0.066	−0.126
*p* value	0.2197	0.0011	0.1906	0.0311
Total protein				
Correlation	−0.023	0.037	−0.036	0.006
*p* value	0.6425	0.4564	0.4709	0.9185
Creatinine				
Correlation	0.171	0.015	0.108	0.164
*p* value	0.0006	0.7646	0.0305	0.0047
25(OH)D				
Correlation	0.170	−0.049	0.055	0.051
*p* value	0.0006	0.3240	0.2766	0.3853
Vitamin B12				
Correlation	0.074	0.010	−0.011	0.040
*p* value	0.1398	0.8387	0.8320	0.4987
Zinc				
Correlation	−0.051	−0.152	−0.062	−0.059
*p* value	0.3518	0.0054	0.2617	0.3644
Corrected calcium				
Correlation	0.040	0.120	−0.048	0.072
*p* value	0.4220	0.0167	0.3414	0.2213
Serum ferritin				
Correlation	−0.018	−0.043	0.093	0.067
*p* value	0.7183	0.3885	0.0622	0.2530
Hemoglobin				
Correlation	−0.150	0.040	0.156	−0.011
*p* value	0.0027	0.4241	0.0017	0.8576

25(OH)D, 25-hydroxyvitamin D. * Pearson correlation coefficient.

**Table 4:** **a** Comparison of nutritional biomarker levels with supplement use. **b** Comparison of nutritional biomarker levels with supplement use by gender.

**(a)**							
**Nutritional Biomarkers**	**Total**	**Non-Supplement Users**			**Supplement Users**		***p* Value ^a^**
Corrected calcium (mmol/L)	2.232 (0.004)	2.230 (0.004)			2.240 (0.010)		0.2965
25(OH)D (μg/L)	30.42 (0.51)	29.49 (0.56)			34.32 (1.06)		0.0002
Vitamin B12 (pmol/L)	423.4 (9.7)	393.6 (9.4)			600.5 (26.8)		<0.0001
Serum ferritin (μg/L)	249.20 (7.95)	249.04 (8.02)			254.97 (59.15)		0.9030
Hemoglobin (g/dL)	13.73 (0.07)	13.78 (0.07)			12.12 (0.46)		<0.0001
**(b)**							
**Nutritional Biomarkers**	**Total**	**Non-Supplement Users Men**	**Non-Supplement Users Women**	***p* Value ^a^**	**Supplement Users Men**	**Supplement Users Women**	***p* Value ^a^**
Corrected calcium (mmol/L)	2.232 (0.004)	2.210 ± 0.006	2.249 ± 0.006	<0.0001	2.224 ± 0.014	2.249 ± 0.013	0.2477
Total participants (*n*)	400	153	159		30	58	
25(OH)D (μg/L)	30.42 (0.51)	31.25 ± 0.84	27.78 ± 0.73	0.0020	35.96 ± 2.15	33.57 ± 1.19	0.2985
Total participants (*n*)	400	159	164		24	53	
Vitamin B12 (pmol/L)	423.4 (9.7)	369.8 ± 11.2	414.1 ± 14.5	0.0192	560.8 ± 43.3	629.3 ± 33.7	0.2103
Total participants (*n*)	396	157	182		24	33	
Serum ferritin (μg/L)	249.20 (7.95)	281.27 ± 13.34	222.40 ± 9.28	0.0002	339.17 ± 74.44	107.63 ± 36.03	0.0532
Total participants (*n*)	400	176	213		7	4	
Hemoglobin (g/dL)	13.73 (0.07)	14.41 ± 0.09	13.25 ± 0.07	<0.0001	12.40 ± 0.71	11.63 ± 0.29	0.4492
Total participants (*n*)	400	176	213		7	4	

25(OH)D, 25-hydroxyvitamin D. Values presented are mean (SE). ^a^ Analysis of variance; *p* value significance < 0.05.

**Table 5:** Factors associated with nutritional biomarkers.

**(a)**												
**Parameters**	**Pre-Albumin (mg/dL)**			**Albumin (mg/dL)**			**Total Protein (g/L)**			**Creatinine (μmol/L)**		
	**Estimate**	**SE**	***p* Value**	**Estimate**	**SE**	***p* Value**	**Estimate**	**SE**	***p* Value**	**Estimate**	**SE**	***p* Value**
Intercept	42.1	5.78	<0.0001	50.5	2.2	<0.0001	72.2	3.1	<0.0001	−0.81	16.7	0.9611
Gender												
Male	2.82	0.63	<0.0001	0.38	0.29	0.1880	-0.32	0.46	0.4909	31.2	2.5	<0.0001
Female (reference)	Reference			Reference			Reference			Reference		
Age (year)	−0.14	0.057	0.0145	−0.026	0.027	0.3329	−0.0034	0.043	0.9363	0.92	0.23	0.0001
BMI (kg/m^2^)	0.18	0.12	0.1115	−0.12	0.046	0.0113	-	-	-	-	-	-
Calf circumference (cm)	−0.31	0.12	0.0106	-	-	-	-	-	-	-	-	-
C-reactive protein (mg/L)	−0.17	0.047	0.0004	−0.043	0.023	0.0601	-	-	-	0.4	0.2	0.0423
Ethnicity												
Chinese	-	-	-	-	-	-	-	-	-	-	-	-
Non-Chinese (reference)	-	-	-	-	-	-	-	-	-	-	-	-
Nutrition literacy score												
2 or 3	-	-	-	-	-	-	-	-	-	-	-	-
4 or 5	-	-	-	-	-	-	-	-	-	-	-	-
6 or 7 (reference)	-	-	-	-	-	-	-	-	-	-	-	-
Education												
No formal education or primary level	-	-	-	-	-	-	-	-	-	-	-	-
Secondary O/N level or equivalent	-	-	-	-	-	-	-	-	-	-	-	-
A level or equivalent	-	-	-	-	-	-	-	-	-	-	-	-
University and above (reference)	-	-	-	-	-	-	-	-	-	-	-	-
Housing type												
HDB 1–3 room flats	-	-	-	-	-	-	-	-	-	-	-	-
HDB 4–5 room flats	-	-	-	-	-	-	-	-	-	-	-	-
Private properties + others	-	-	-	-	-	-	-	-	-	-	-	-
Vitamin D supplement use												
No	-	-	-	-	-	-	-	-	-	-	-	-
Yes (reference)	-	-	-	-	-	-	-	-	-	-	-	-
Vitamin B12 supplement use												
No	-	-	-	-	-	-	-	-	-	-	-	-
Yes (reference)	-	-	-	-	-	-	-	-	-	-	-	-
Iron supplement use												
No	-	-	-	-	-	-	-	-	-	-	-	-
Yes (reference)	-	-	-	-	-	-	-	-	-	-	-	-
**(b)**												
**Parameters**	**25(OH)D (μg/L)**				**Vitamin B12 (pmol/L)**				**Zinc (μg/L)**			
	**Estimate**		**SE**	***p* Value**	**Estimate**	**SE**	***p* Value**		**Estimate**	**SE**	***p* Value**	
Intercept	5.07		7.54	0.5020	616	139.6	<0.0001		1057.3	126	<0.0001	
Gender												
Male	2.56		1.07	0.0179	−23.2	20	0.2462		2.9	16.2	0.8600	
Female (reference)	Reference				Reference				Reference			
Age (year)	0.31		0.1	0.0017	−0.089	1.9	0.9619		−1.3	1.4	0.3752	
BMI (kg/m^2^)	-		-	-	-	-	-		−5.6	2.6	0.0343	
Calf circumference (cm)	-		-	-	-	-	-		-	-	-	
C-reactive protein (mg/L)	-		-	-	-	-	-		-	-	-	
Ethnicity												
Chinese	5.69		1.44	<0.0001	-	-	-		38.1	21.9	0.0829	
Non-Chinese (reference)	Reference				-	-	-		Reference			
Nutrition literacy score												
2 or 3	-		-	-	−164.2	83.7	0.0509		-	-	-	
4 or 5	-		-	-	−31.7	19.9	0.1125		-	-	-	
6 or 7 (reference)	-		-	-	Reference				-	-	-	
Education												
No formal education or primary level	-		-	-	-	-	-		-	-	-	
Secondary O/N level or equivalent	-		-	-	-	-	-		-	-	-	
A level or equivalent	-		-	-	-	-	-		-	-	-	
University and above (reference)	-		-	-	-	-	-		-	-	-	
Housing type												
HDB 1–3 room flats	-		-	-	-	-	-		-	-	-	
HDB 4–5 room flats	-		-	-	-	-	-		-	-	-	
Private properties + others	-		-	-	-	-	-		-	-	-	
Vitamin D supplement use												
No	−3.89		1.32	0.0036	-	-	-		-	-	-	
Yes (reference)	Reference				-	-	-		-	-	-	
Vitamin B12 supplement use												
No	-		-	-	−195.7	28.8	<0.0001		-	-	-	
Yes (reference)	-		-	-	Reference				-	-	-	
Iron supplement use												
No	-		-	-	-	-	-		-	-	-	
Yes (reference)	-		-	-	-	-	-		-	-	-	
**(c)**												
**Parameters**	**Corrected Calcium (mmol/L)**				**Serum Ferritin (μg/L)**				**Hemoglobin (g/dL)**			
	**Estimate**		**SE**	***p* Value**	**Estimate**	**SE**	***p* Value**		**Estimate**	**SE**	***p* Value**	
Intercept	2.089		0.074	<0.0001	154.42	125.36	0.2190		11.8	1.2	<0.0001	
Gender												
Male	−0.043		0.0095	<0.0001	85.46	18.99	<0.0001		1.2	0.14	<0.0001	
Female (reference)	Reference				Reference				Reference			
Age (year)	0.0016		0.00086	0.0580	–0.13	1.73	0.9382		−0.03	0.013	0.0212	
BMI (kg/m^2^)	0.0027		0.0015	0.0718	-	-	-		0.048	0.022	0.0273	
Calf circumference (cm)	-		-	-	-	-	-		-	-	-	
C-reactive protein (mg/L)	-		-	-	-	-	-		-	-	-	
Ethnicity												
Chinese	−0.023		0.013	0.0720	78.88	24.82	0.0016		0.41	0.19	0.0274	
Non-Chinese (reference)	Reference				Reference				Reference			
Nutrition literacy score												
2 or 3	-		-	-	-	-	-		-	-	-	
4 or 5	-		-	-	-	-	-		-	-	-	
6 or 7 (reference)	-		-	-	-	-	-		-	-	-	
Education												
No formal education or primary level	-		-	-	−31.57	34.12	0.3555		0.17	0.25	0.4943	
Secondary O/N level or equivalent	-		-	-	27.04	28.23	0.3391		−0.11	0.21	0.6055	
A level or equivalent	-		-	-	−3.87	31.13	0.9012		0.37	0.23	0.1131	
University and above (reference)	-		-	-	Reference				Reference			
Housing type												
HDB 1–3 room flats	-		-	-	-	-	-		-	-	-	
HDB 4–5 room flats	-		-	-	-	-	-		-	-	-	
Private properties + others	-		-	-	-	-	-		-	-	-	
Vitamin D supplement use												
No	-		-	-	-	-	-		-	-	-	
Yes (reference)	-		-	-	-	-	-		-	-	-	
Vitamin B12 supplement use												
No	-		-	-	-	-	-		-	-	-	
Yes (reference)	-		-	-	-	-	-		-	-	-	
Iron supplement use												
No	-		-	-	-	-	-		1.96	0.42	<0.0001	
Yes (reference)	-		-	-	-	-	-		Reference			

## Supplementary Materials

The following are available online at https://www.mdpi.com/2072-6643/12/11/3329/s1, Table S1: Reference ranges of nutritional biomarkers in this study; Table S2: Factors associated with serum 25(OH)D and zinc categorized by the status.
