# Supplementary material for: Nutritional Biomarkers and Associated Factors in Community-Dwelling Older Adults: Findings from the SHIELD Study
Source: Nutrients. 2020 Oct 29;12(11):3329. doi: 10.3390/nu12113329 (PMC7693785; doi:10.3390/nu12113329)
Supplement: Supplementary file 1 [file nutrients-12-03329-s001.pdf]

Table S1. Reference ranges of nutritional biomarkers in this study.

Nutritional biomarkers	Low	Normal	High
Pre-albumin (mg/dL)	<20	20–40	>40
Albumin (mg/dL)	<37	37–51	>51
Total protein (g/L)	<62	62–82	>82
Creatinine (μmol/L)			
Men	<65	65–125	>125
Women	<50	50–90	>90
25(OH)D (μg/L)	<30	30–100	>100
Vitamin B12 (pmol/L)	<132	132–835	>835
Zinc (μg/L)	<724	724–1244	>1244
Corrected calcium (mmol/L)	<2.10	2.10–2.60	>2.60
Serum ferritin (μg/L)			
Men	<32	32–294	>294
Women	<18.2	18.2–339	>339
Hemoglobin (g/dL)	<11.5	11.5–15.0	>15.0

Table S2. Factors associated with serum 25(OH)D and zinc categorized by the status*

Variables	25(OH)D				Zinc		
	**Deficient** (<20 μg/L) n = 54	**Insufficient** (20–29 μg/L) n = 154	**Sufficient** (≥30 μg/L) n = 192	***p* value**[a]	**Low** (<724 μg/L) n = 34	**Normal/High** (≥724 μg/L) n = 298	***p* value**[a]
Age (year)	70.3 (0.7)	70.3 (0.4)	72.2 (0.4)	0.0013	72.0 (0.9)	71.2 (0.3)	0.4009
Gender, n (%)							
Male	17 (31)	71 (46)	95 (49)	0.0633	16 (47)	133 (45)	0.8563
Female	37 (69)	83 (54)	97 (51)		18 (53)	165 (55)	
Body weight (kg)	59.65 (1.44)	63.02 (0.82)	61.56 (0.64)	0.0740	64.8 (1.76)	61.3 (0.54)	0.0398
BMI (kg/m^2)	24.26 (0.54)	24.94 (0.25)	24.28 (0.2)	0.1120	25.61 (0.51)	24.30 (0.17)	0.0164
Mid upper arm circumference (cm)	27.22 (0.48)	27.88 (0.27)	27.75 (0.23)	0.4431	28.74 (0.63)	27.58 (0.19)	0.0519
Calf circumference (cm)	34.22 (0.46)	35.75 (0.26)	35.13 (0.23)	0.0083	36.29 (0.42)	35.16 (0.19)	0.0481
Fat mass (kg)	18.01 (1.02)	18.67 (0.51) [b]	17.19 (0.44)	0.1038	19.06 (1.26)	17.58 (0.35) [d]	0.1892
Fat %	30.02 (1.33)	29.04 (0.7) [b]	27.63 (0.63)	0.1374	29.12 (1.79)	28.33 (0.50) [d]	0.6212
Total body skeletal muscle mass (kg)	39.38 (1.19)	41.84 (0.67) [b]	41.97 (0.58)	0.1109	43.3 (1.73)	41.36 (0.47) [d]	0.2007
Bone mass (kg)	2.26 (0.06)	2.41 (0.03) [b]	2.4 (0.03)	0.0682	2.46 (0.09)	2.38 (0.02) [d]	0.2967
C-reactive protein (mg/L)	2.95 (0.59) [c]	2.17 (0.23) [c]	2.61 (0.77) [c]	0.7779	3.29 (0.75) [e]	2.35 (0.50) [e]	0.5085
Physical Activity Scale for the Elderly score	110.1 (9.2)	117.5 (5.3)	123.6 (4.4)	0.3427	113.8 (10.5)	121.2 (3.8)	0.5296
Modified Barthel Index score	99.2 (0.4)	99.4 (0.2)	99.8 (0.1)	0.2190	97.7 (1.0)	99.7 (0.1)	<0.0001
Total Charlson Comorbidity Score	0.04 (0.03)	0.05 (0.02)	0.01 (0.01)	0.3080	0.1 (0.1)	0.01 (0.005)	<0.0001
Ethnicity, n (%)							
Chinese	34 (63)	128 (83)	170 (89)	0.0002	23 (68)	253 (85)	0.0161
Non-Chinese	20 (37)	26 (17)	22 (11)		11 (32)	45 (15)	
Nutrition literacy score, n (%)							
2 or 3	1 (2)	2 (1)	2 (1)	0.3672	0	5 (2)	0.0726
4 or 5	29 (54)	62 (40)	89 (46)		21 (62)	122 (41)	
6 or 7	24 (44)	90 (58)	101 (53)		13 (38)	171 (57)	
Marital status, n (%)							
Single	1 (2)	12 (8)	24 (13)	0.0668	5 (15)	30 (10)	0.5162
Married	40 (74)	122 (79)	135 (70)		22 (65)	223 (75)	
Separated	1 (2)	1 (1)	0 (0)		0 (0)	1 (<1)	

Divorced	4 (7)	5(3)	8 (4)		2 (6)	11 (4)	
Widowed	8 (15)	14 (9)	25 (13)		5 (15)	33 (11)	
Education, *n* (%)							
No formal education/primary	10 (19)	24 (16)	28 (15)	0.6474	10 (29)	38 (13)	0.0987
Secondary; O/N level	29 (54)	72 (47)	85 (44)		12 (35)	141 (47)	
A level; Polytechnic, diploma and other professional qualifications	10(19)	41 (27)	49 (26)		8 (24)	82 (28)	
University and above	5 (9)	17 (11)	30 (16)		4 (12)	37 (12)	
Housing type, *n* (%)							
HDB 1-3 room flats	15 (28)	27 (18)	32 (17)	0.0805	10 (29)	53 (18)	0.2085
HDB 4-5 room flats	28 (52)	74 (48)	84 (44)		15 (44)	135 (45)	
Private properties + others	11 (20)	53 (34)	76 (40)		9 (26)	110 (37)	
Smoking, *n* (%)							
Non-smoker	45 (83)	133 (86)	155 (81)	0.4765	23 (68)	255 (86)	0.0097
Daily/occasional smoker	2 (4)	4 (3)	4 (2)		0 (0)	7 (2)	
Past smoker	7 (13)	17 (11)	33 (17)		11 (32)	36 (12)	
Drinking habit, *n* (%)							
No alcohol	48 (89)	109 (71)	130 (68)	0.0359	27(79)	209 (70)	0.2672
< once a month	4 (7)	29 (19)	36 (19)		6 (18)	53 (18)	
≥ once a month	2 (4)	16 (10)	26 (14)		1 (3)	36 (12)	
Hospital admission in the last 6 months, *n* (%)							
Yes	4 (7)	9 (6)	5 (3)	0.1523	4 (12)	9 (3)	0.0338
No	50 (93)	145 (94)	187 (97)		30 (88)	289 (97)	
Supplement use[f]							
Yes	1 (2)	26 (17)	50 (26)	<0.0001	1 (3)	1 (<1)	0.1946
No	53 (98)	128 (83)	142 (74)		33 (97)	297 (>99)	
Use of ONS, *n* (%)							
Yes	8 (15)	25 (16)	34 (18)	0.8826	7 (21)	52 (17)	0.6382
No	46 (85)	129 (84)	158 (82)		27 (79)	246 (83)	
Eating less due to reduced taste sensation, *n* (%)							
Yes	2 (4)	5 (3)	5 (3)	0.7808	5 (15)	4 (1)	0.0008
No	52 (96)	149 (97)	187 (97)		29 (85)	294 (99)	
Eating less due to poor vision, *n* (%)							
Yes	2 (4)	0 (0)	1 (1)	0.0491	2 (6)	0 (0)	0.0102
No	52 (96)	154 (100)	191 (99)		32 (94)	298 (100)	

Ability to shop, cook and feed oneself, *n* (%)							
Yes	48 (89)	150 (97)	187 (97)	0.0224	30 (88)	288 (97)	0.0438
No	6 (11)	4 (3)	5 (3)		4 (12)	10 (3)	

25(OH)D, 25-hydroxyvitamin D. BMI, body mass index. O/N level, Ordinary/Normal level. A level, Advanced level. HDB, housing development board. * Values presented are mean (SE) for continuous variables, and *n* (percentage) for categorical variables. [a]Analysis of variance (continuous variables); Fisher's Exact test (categorical variables). *p*-value significance < 0.05. [b]*n* = 152, [c]*n* = 30 (deficient), *n* = 128 (insufficient), *n* = 136 (sufficient), [d]*n* = 297, [e]*n* = 27 (low), *n* = 215 (normal/high). [f]Vitamin D supplement use for 25(OH)D analysis. Zinc supplement use for serum zinc analysis.
